# Postmortem Diagnosis of Invasive Meningococcal Disease

**DOI:** 10.3201/eid2003.131245

**Published:** 2014-03

**Authors:** Alison D. Ridpath, Tanya A. Halse, Kimberlee A. Musser, Danielle Wroblewski, Christopher D. Paddock, Wun-Ju Shieh, Melissa Pasquale-Styles, Irini Scordi-Bello, Paula E. Del Rosso, Don Weiss

**Affiliations:** Centers for Disease Control and Prevention, Atlanta, Georgia, USA (A.D. Ridpath, C.D. Paddock, W.-J. Shieh);; New York City Department of Health and Mental Hygiene, New York, New York, USA (A.D. Ridpath, P.E. Del Rosso, D. Weiss);; Wadsworth Center, New York State Department of Health, Albany, New York, USA (T.A. Halse, K.A Musser, D. Wroblewski);; New York City Office of the Chief Medical Examiner, New York (M. Pasquale-Styles, I. Scordi-Bello)

**Keywords:** *Neisseria meningitidis*, New York City, polymerase chain reaction, PCR, bacteria, New York

## Abstract

We diagnosed invasive meningococcal disease by using immunohistochemical staining of embalmed tissue and PCR of vitreous humor from 2 men in New York City. Because vitreous humor is less subject than other body fluids to putrefaction, it is a good material for postmortem analysis.

Invasive meningococcal disease is nationally reportable in the United States, and its case-fatality rate is 10%–14% (*1*). In New York City, New York, USA, every suspected or confirmed case is investigated to rapidly identify and recommend antimicrobial prophylaxis to close contacts. The risk for disease is highest during the initial 1–4 days after exposure. Fatal invasive meningococcal disease may go undiagnosed, which impairs prevention efforts and understanding of transmission. We describe 2 cases of serogroup C meningococcal disease diagnosed post mortem by PCR from vitreous humor and immunohistochemical (IHC) staining of tissues collected at autopsy.

## The Cases

### Case 1

In the fall of 2012, a man in his early 30s was found dead in his room by his family. He had attended a party and the next morning reported fever, chills, and general malaise (day 1). He was not seen again until day 3 when a family member found him unresponsive in his room. Emergency medical services personnel pronounced him dead. The case was reported to the New York City Office of the Chief Medical Examiner, and the medicolegal investigator noted that the man was in an early state of decomposition. No suspicious circumstances or evidence of external trauma or alcohol or drug use were present.

**Figure 1:**
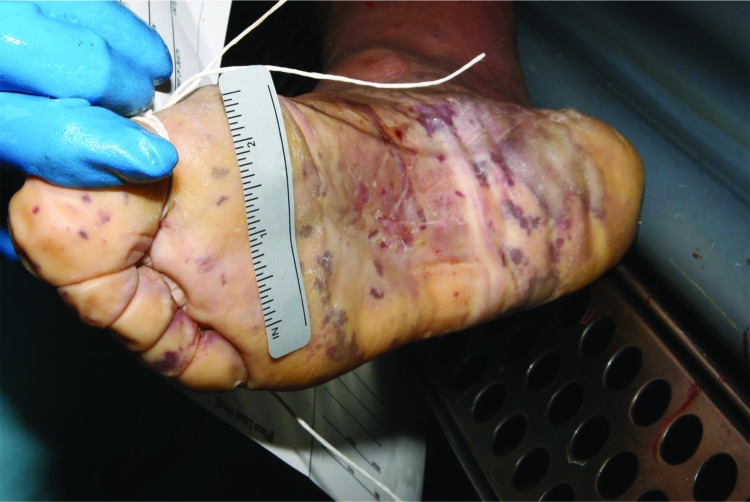
Postmortem purpuric rash on sole of a man for whom invasive meningococcal disease was diagnosed after death (case 1), New York City, New York, USA.

An autopsy performed on day 4 was remarkable for a purpuric rash more pronounced on the legs, arms, hands, and soles of feet (Figure 1). Early decomposition, commented on by the medicolegal investigator, was also noted at autopsy. Internal organs had a markedly soft consistency, but no other substantial abnormality was noted. The heart and valves were within normal limits without evidence of vegetation. The only substantial internal finding was a thin layer of purulent exudate on the leptomeninges. Cerebrospinal fluid (CSF) and blood samples could not be obtained. A limited amount of vitreous humor was collected; brain and other organ tissue samples collected were fixed in formalin.

The body was brought to the funeral home and embalmed on day 6. The medical examiner reviewing the case on day 6 became concerned that the patient might have died of meningococcal disease, and the examiner notified the New York City Department of Health and Mental Hygiene (DOHMH). DOHMH recommended that the medical examiner collect additional samples, specifically skin, for IHC. Three hours after embalming, skin samples were collected from areas of purpuric rash on the leg and stored in saline. Vitreous humor and skin samples were sent to Wadsworth Center laboratory of the New York State Department of Health (Albany, NY, USA) for testing by PCR, and tissue specimens (brain, lung, heart, liver, and kidney collected at autopsy and fixed in formalin and skin collected after embalming) were sent to the Centers for Disease Control and Prevention (CDC; Atlanta, GA, USA) for IHC staining and PCR (*2*–*4*).

Skin and vitreous humor specimens were positive for *Neisseria meningitidis* serogroup C DNA by real-time PCR at Wadsworth Center (*2*). Gram-staining of brain and skin tissue (examination performed at CDC) showed gram-negative cocci and gram-positive bacilli in leptomeninges and vascular lumens. Widespread gram-positive bacilli were present in brain parenchyma without corresponding inflammation. Therefore, the gram-positive bacilli were deemed likely to represent postmortem bacteria overgrowth and did not contribute to an etiologic diagnosis. IHC assays that used a polyclonal anti–*N. meningitidis* group Y antiserum that is broadly reactive with serogroups A, B, C, W, and Y, and a specific monoclonal anti–*N. meningitidis* serogroup C antibody, revealed immunostaining in the leptomeninges, lung, and skin tissues (*3*,*4*). Results confirmed *N. meningitidis* serogroup C as the etiologic agent causing acute meningitis and systemic infection.

Clinical, PCR, and IHC findings were all consistent with invasive meningococcal disease. All close contacts of the patient were located and given prophylaxis. No secondary cases were identified.

### Case 2

Approximately 2 months after the first case occurred, a man in his mid-30s was found dead in his apartment by his friends and police after he was reported missing from work. He had last spoken to friends 4 days previously when he reported a sore throat (day 1). The man had a history of HIV infection and crystal methamphetamine use, which was confirmed by urine toxicology at autopsy.

Autopsy was performed on day 6. The patient had marked putrefactive skin changes on the left side of the body, consistent with his postmortem position, and visible purpura on the right side of the torso and lower extremities but not on palms or soles. Flattening of cerebral gyri with apparent focal subarachnoid purulent exudate was also reported. Cerebral cortex and leptomeninge tissues were cultured and grew gram-positive rods and mixed flora. Tissue samples of lung, liver, spleen, kidney, and pancreas were fixed in formalin and sent to CDC. Vitreous humor and a swab of the leptomeninges collected at autopsy were sent to Wadsworth Center.

**Figure 2:**
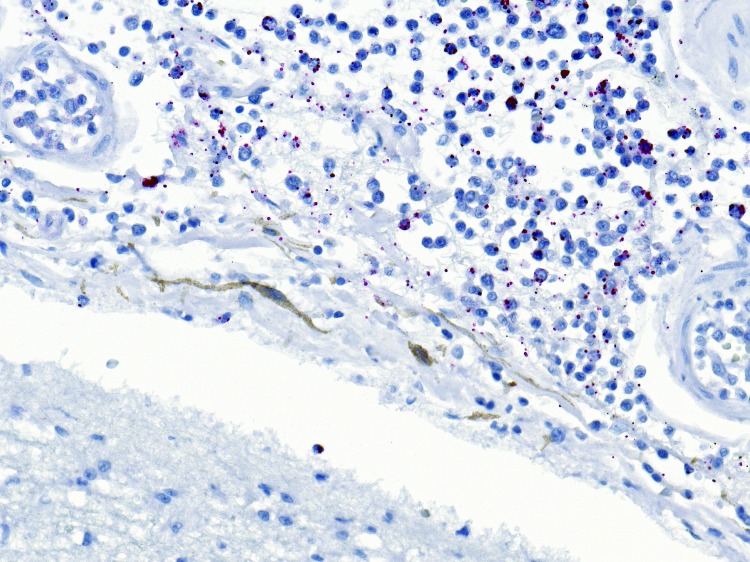
Immunostaining of *Neisseria menigitidis* in meninges of a man for whom invasive meningococcal disease was diagnosed after death (case 2), New York City, New York, USA. Naphthol fast red substrate with light hematoxylin counterstain. Original magnification ×25.

At Wadsworth Center, testing of the vitreous humor by real-time PCR showed *N. meningitidis* serogroup C DNA, *Haemophilus influenzae* DNA, *Streptococcus agalactiae* DNA, and *Staphylococcus aureus* DNA (*2*). As in case 1, the bacteria other than *N. meningitidis* were thought to represent postmortem bacteria overgrowth and did not contribute to etiologic diagnosis. At CDC, *N*. *meningitidis* DNA was extracted and amplified by PCR from formalin-fixed paraffin-embedded brain, liver, lung, spleen, and kidney tissue (*4*). Although multiple tissues showed marked autolysis, distinct and specific IHC staining for *N. meningitidis* serogroup C was identified within blood vessels and in neutrophilic infiltrates in the leptomeninges (Figure 2) (*3*,*4*). The results confirmed *N. meningitidis* serogroup C as the etiologic agent causing acute meningitis and systemic infection. DOHMH did not learn about the case until after it was too late to administer prophylaxis to close contacts, but no known secondary cases were identified.

## Conclusions

Suspecting and diagnosing meningococcal disease early is critical for initiating timely prophylaxis and preventing secondary cases. The 2 cases described here posed multiple challenges for diagnosing invasive meningococcal disease. In both cases, CSF and blood samples, where *N. meningitidis* is typically identified, were unavailable. Given the patients’ unattended deaths and delayed discovery, putrefaction had set in and tissue samples were fairly decomposed. Additional bacteria were identified through real-time PCR or culture, further complicating the diagnosis for both cases.

*N. meningitidis* is a fastidious organism that frequently undergoes autolysis, although it has been found in CSF by PCR up to 10 days postmortem (*5*,*6*). Vitreous fluid has been described as a useful specimen for postmortem analysis because the eye is isolated and the fluid is less subjected to contamination or purification (*7*). *N. meningitidis* has been isolated from vitreous humor of living patients, usually in conjunction with symptoms of meningococcal endopthalmitis (*8*,*9*). We have demonstrated that postmortem diagnosis of *N. meningitidis* from vitreous humor and IHC staining <3 days after death is possible. This testing might prove a useful option for medical examiners and public health officials for diagnosing suspected meningococcal disease when blood and CSF are unavailable for testing.
